# The Voluntary Hospitals

**Published:** 1903-02-14

**Authors:** 


### THE VOLUNTARY HOSPITALS.

FROM the *Daily Chronicle* of Friday, the 6th inst., we take the following reports of two coroner's inquests held during the current week in London. We dealt with the points raised by the first case under the heading "Hospital Questions which Press" on pp. 327–8 of our last issue. The matter is also dealt with in our leading columns this week.

### A CORONER ON THE NEED FOR POPULAR CONTROL.

An inquest was held at Westminster yesterday by Mr. John Troutbeck concerning the death of Edward Evans, aged 52 years, who died in the Poland Street Workhouse.

On the evening of January 27 the deceased was knocked down by a cab. He was taken by a policeman to the Middlesex Hospital, where two young surgeons, it was said, examined him, and said they were unable to say what was the matter with him, at the same time telling the officer to take the man to the Poland Street Workhouse. After being in the hospital not more than fifteen minutes the constable walked with the deceased to the workhouse, where the injured man was put in the insane ward, as was usual with all obstreperous patients. Death took place on Monday. A post-mortem showed that the skull was fractured, and that death was due to shock.

The Coroner, in summing up, said it was perfectly obvious that when an injured man was taken to a hospital he should be seen by a surgeon of experience, and it was only prudent to await developments. Although it was not suggested that the deceased's removal accelerated his death, yet he thought the jury were quite right to take notice of these things, for it was likely to be productive of much good. It stimulated public opinion with regard to hospitals. The subject was now engaging much attention, and the trend of public opinion was that there should be further popular control of hospitals. Popular control they already possessed in a very small degree through jurymen at inquests, but he had long felt that that was not sufficient, and they should make as much as they could of the opportunity.

In returning a verdict of accidental death the jury added a rider to the effect that the deceased ought to have been detained at the hospital for a sufficient time to see whether his symptoms developed.

### ADMISSION TO ST. THOMAS'S.

Mr. John Troutbeck last night held an inquiry at Lambeth relative to the death of Mary Gosbee, aged fifty-four years, who died in the Lambeth Infirmary.

She was burnt in consequence of a lamp accident, and was taken to St. Thomas's Hospital, where her burns were dressed, and she was told to attend as an out-patient. Her condition had become so bad by the 19th that she was sent to the Lambeth Infirmary.

Mr. Anthony Bradford, assistant house surgeon at St. Thomas's Hospital, said the deceased was brought there suffering from burns extending from the shoulder down to the wrist. He dressed the injuries, and she came to the hospital twice afterwards. Witness had no power to admit her, and he did not consider it necessary to do anything in the way of examination beyond taking the woman's temperature, unless something beyond the burn was apparent.

Dr. Ludwig Freyberger said that on making a post-mortem he found an extensive burn from the rest of the shoulder, affecting the whole of the skin of the arm, and extending downwards to the edge of the breast. Death was due to shock and exhaustion following the burns in a person suffering from chronic valvular disease of the heart and chronic kidney disease. A woman in that condition required constant nursing, and could not be properly treated in a poor home.

In summing up the Coroner said he considered that there was something in this case which required explanation. When a great public trust like St. Thomas's Hospital had to be administered, it was important to know what was done when a poor person who had no means at home came there for treatment. He thought it was practically admitted that the deceased would have been taken in had her real condition been ascertained at the time, but it was not ascertained. There was no legal right to claim admission to St. Thomas's Hospital, but its conduct was just as liable to criticism as any other institution. It was one of the penalties and one of the advantages of such institutions to be criticised.

The jury returned a verdict of accidental death, and expressed it as their opinion that the question of admitting patients to St. Thomas's Hospital should be placed in more experienced hands.

